# Etude de la qualité bactériologique de l’eau utilisée dans l’industrie agroalimentaire dans le Nord du Maroc

**DOI:** 10.11604/pamj.2017.26.13.10591

**Published:** 2017-01-05

**Authors:** El Houcine Haijoubi, Fatiha Benyahya, Abdrezzak Bendahou, Faima Zahra Essadqui, Mohammed El Behhari, Ahmed Fouad El Mamoune, Naima Nourouti Ghailani, Mohcine Bennani Mechita, Amina Barakat

**Affiliations:** 1 Laboratoire de Génomique Humain, Faculté des Sciences et Techniques de Tanger, Maroc; 2 Institut Pasteur du Maroc à Tanger

**Keywords:** Qualité bactériologique, eau de puits, eau de réseau public, industrie agroalimentaire, Bacterial quality, wells water, public-supply water, agri-food industry

## Abstract

**Introduction:**

L'eau est utilisée d'une façon primordiale dans tout le processus de la fabrication des produits alimentaires. Les industries agroalimentaires du Nord du Maroc utilisent différentes sources d'eaux mais l'eau de réseau public et l'eau de puits sont les principales sources d'eau utilisée. Cette eau peut s'avérer la source principale des éventuelles contaminations et altérations des aliments. Notre but est d'évaluer la qualité bactériologique de l'eau utilisée par les industries agroalimentaires dans la région du Nord du Maroc, d'identifier les différents germes responsables de la pollution de ces eaux et de définir les principales causes de cette pollution.

**Méthodes:**

Des échantillons d'eau prélevés aux robinets ou des puits ont été analysés pour la recherche des germes indicateurs de la pollution (coliformes totaux (CT), coliformes fécaux (CF), entérocoques intestinaux (E), microorganismes revivifiables (MOR), anaérobies sulfitoréducteurs) et les germes pathogènes (Salmonelles, Staphylocoques, *Pseudomonas aeruginosa*). Le dénombrement des bactéries a été fait par la technique de filtration et par incorporation en milieu solide en surfusion.

**Résultats:**

Les résultats ont montré que les eaux du réseau public ont été de qualité bactériologique satisfaisante tandis que 40% des eaux des puits ont été non conformes aux normes à cause de la présence des indicateurs de pollution CT, CF, E et MOR. En revanche, les germes pathogènes, en particulier les Salmonelles, ont été absents dans les eaux de tous les puits analysés.

**Conclusion:**

La pollution de ces puits a été généralement liée au non-respect des conditions de puisage hygiéniques. La qualité bactériologique des eaux de ces puits peut être améliorée par une protection adéquate.

## Abstract (en)

**Introduction:**

Water is used predominantly in food manufacturing process. Northern morocco agro-food industries use different sources of water, but public water and wells water are the main sources of water used. This water can be the main source of possible food contaminations and alterations. This study aims is to assess the bacteriological quality of water used in the agro-food industries in the Northern region of Morocco, to identify the different types of germs responsible for the pollution of these waters and to establish the main causes of this pollution.

**Methods:**

Water samples taken from taps or wells were analyzed to detect pollution indicator germs (total coliform (TC), fecal coliform (FC), intestinal enterococci (E), revivable microorganisms (RM), sulphite-reducing anaerobes) and pathogens (Salmonella, Staphylococci, Pseudomonas aeruginosa). The enumeration of the bacteria was performed by filtration technique and incorporation obtained through supercooled solid state.

**Results:**

The results showed that public-supply waters were of satisfactory bacteriological quality while 40% of the wells water was non-compliant with water quality standards due to the presence of TC, FC, E and RM pollution indicators. In contrast, pathogens, particularly Salmonellae, were absent in all the wells water analyzed.

**Conclusion:**

Well water pollution was generally due to failure to meet hygienic requirements for water pumping. Bacteriological quality of these wells water could be improved by adequate protection.

## Introduction

Le contexte économique mondial actuel entraîne une concurrence très vive entre les entreprises agroalimentaires. Celles-ci doivent impérativement satisfaire leurs clients et proposer des produits de qualité. Cette qualité constitue de plus en plus une préoccupation essentielle de la société de consommation. La qualité des produits agroalimentaires peut être altérée à cause des contaminations par des germes. Au cours de tous le processus de la fabrication de ces produits, l´eau est utilisée d'une façon primordiale. Cette eau peut s´avérer la source principale des éventuelles contaminations et altérations lorsque les conditions d´hygiène, de salubrité et de bonnes pratiques ne sont pas respectées. Ainsi, l'industrie agroalimentaire doit prendre toutes les mesures possibles pour s´assurer en tout temps que l´eau est salubre. L'évaluation de la qualité de l'eau consiste en un dénombrement des bactéries indicatrices d'une contamination d'origine fécale ou en une détection de la présence des bactéries pathogènes en utilisant des méthodes normalisées ou validées de microbiologie classique [1, 2]. Notre présente étude a visé à déterminer la qualité bactériologique de l'eau utilisée dans le processus de fabrication des aliments dans les industries agroalimentaires du Nord du Maroc.

## Méthodes

**Echantillonnage:** deux cent trente-quatre (234) échantillons d'eau ont été prélevés dans 30 entreprises d´industrie agroalimentaire du Nord du Maroc. Treize (13) entreprises sont alimentées par l'eau du réseau public, quinze (15) entreprises sont alimentées par d'eau de puits et deux (2) entreprises sont alimentées par l'eau de réseau et l'eau de puits. Les prélèvements d'eau ont été collectés sur une période de 30 mois entre 2011 et 2013. Les prélèvements de l'eau de puits ont été effectués deux fois par ans au cours de la période estivale et la période hivernale. Les prélèvements de l'eau du réseau public ont été réalisés trois fois par ans à raison de deux prélèvements pour chaque industrie l'un à l'entrée du réseau et l'autre à la sortie. Les échantillons représentatifs ont été rassemblés selon les Méthodes Standard pour l'analyse d´Eau et d'Eaux usées dans des récipients stériles et bien identifiés [**3**]. Les échantillons ont été conservés et transportés à une température de +4 à + 8°C. L'analyse bactériologique a été réalisée dans un délai maximal de 8h après le prélèvement.

**Analyse bactériologique:** les paramètres bactériologiques ont été analysés en double exemplaires. Les germes recherché dans les échantillons des eaux analysées sont : les coliformes totaux (CT), les coliformes fécaux (CF), les Entérocoques intestinaux (E), les anaérobies sulfito-réducteurs, Pseudomonas aeruginosa, les micro-organismes revivi-fiables (MOR) et Salmonella selon la méthode standard d'analyse de l'eau et de l'eau usée en utilisant la technique de la concentration par filtration sur membrane (FM) [**3**] et la technique d'incorporation en milieu solide. Le contrôle bactériologique de l'eau a été réalisé selon les normes de potabilité marocaine et AFNOR qui stipulent l'absence totale des germes nuisibles voire les indicateurs de la pollution.

**Mesure des paramètres physicochimiques:** le chlore résiduel a été mesuré par une méthode colorimétrique utilisant l´analyseur de Chlore libre Policontrol ^®^. Les températures d´eau ont été prises utilisant la colonne de mercure et la valeur de pH utilisant le pH-mètre.

## Résultats

### Analyse bactériologique

***Analyse bactériologique de l'eau du réseau public:*** les résultats des analyses bactériologiques des eaux de réseau public utilisées par les entreprises agroalimentaires sujets de notre étude ont montré l'absence totale des germes responsables de la non-conformité des eaux soit à l'entrée ou à la sortie du réseau. Ainsi, ces résultats ont révèlé que ces eaux sont de qualité bactériologique satisfaisante.

***Analyse bactériologique de l'eau de puits:*** les 20 entreprises qui utilisent l'eau de puits comme principale source d'approvisionnement sont localisées dans les régions rurales. Sur ces 20 entreprises, 14 sont des fermes avicoles et 6 ont diverses activités alimentaires. Ces entreprises font des contrôles réguliers de l'eau durant la période des campagnes (estivale et hivernale).

***Analyse bactériologique des eaux utilisées dans les fermes avicoles:*** les résultats des analyses bactériologiques des eaux de puits utilisées dans les fermes avicoles sont consignés dans le **Tableau 1**. Nous avons constaté que la qualité bactériologique de ce type d'eau a été satisfaisante pour 7 puits et non satisfaisante pour les 7 autres puits étudiés. Cette non-conformité est due surtout à la présence des indicateurs de pollution, tels que les coliformes fécaux en particulier l'*E.coli*, les coliformes totaux et les Entérocoques intestinaux. Parmi les 7 puits dont la qualité de l'eau est non satisfaisante, 3 puits ont été contaminés par les coliformes totaux et les coliformes fécaux et les Entérocoques intestinaux, 2 de ces puits ont été contaminés par les coliformes totaux, les coliformes fécaux et 2 puits ont été contaminés par les coliformes totaux et les Entérocoques intestinaux. En plus des germes responsables de la non-conformité (CT, CF et E), nous avons détecté la présence des microorganismes revivifiables (MOR) dans tous les puits contaminés. Les résultats du contrôle de qualité effectués au cours des périodes des compagnes a révélé que la contamination des puits étudiés a été détectée au début de la période hivernal et vers la fin de la période estivale.

**Tableau 1:** Résultats des analyses des prélèvements d’eau de puits pour aviculture

Nombre de puits analysés (14 puits)	Qualité de l’eau	G.R.N.C.
7 puits	Satisfaisant	Néant
3 puits	Non satisfaisant	CT, CF, E, MOR
2 puits	Non satisfaisant	CT, CF, MOR
2 puits	Non satisfaisant	CT, E, MOR

CT: coliformes totaux, CF: coliformes fécaux, E: Entérocoques intestinaux, MOR: microorganismes revivifiables

***Analyse bactériologique des eaux utilisées dans d'autres activités agroalimentaires:*** six (6) entreprises utilisant l'eau de puits dans des activités alimentaires autres que l'aviculture. Les résultats du contrôle de qualité effectués sur les eaux des puits utilisés par ces entreprises ont révélé qu'un seul échantillon sur 6 analysés a été non conforme aux normes en vigueur. Cette non-conformité est due aux indicateurs de la pollution CT, CF et Entérocoques intestinaux généralement responsables de la pollution des eaux.

**Figure 1:**
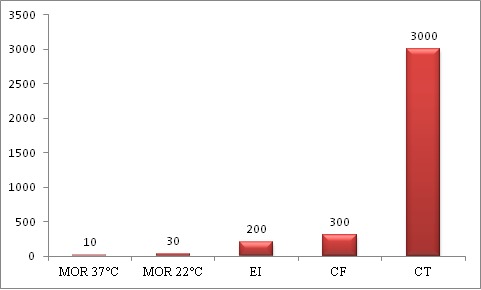
Abondance des germes recherchés dans les eaux des puits en UFC/ 100 ml pour les CT, CF et Entérocoques et en UFC/ ml pour les MOR

***Abondance des germes recherchés dans les eaux des puits:*** l'abondance des germes recherchés dans les eaux de puits analysés au cours de notre étude est représentée sur la **Figure 1** en UFC/ 100 ml pour les CT, CF et Entérocoques et en UFC/ ml pour les MOR. Les résultats de notre étude ont montré l'absence totale dans les puits analysés des germes pathogènes en particulier salmonelles et des bactéries entérites notamment les formes sporulées des sulfito-réducteurs (*Clostridium perfringens*). D'autre part, les résultats ont montré la présence d'un taux très modeste des microorganismes revivifiables (MOR) dénombrés à 22°C et 37°C dans les eaux des puits contaminés.

### Mesure des paramètres physicochimiques

Les valeurs de chlore résiduel mesurées dans les eaux de puits et l'eau de réseau ont respecté la norme établie par la législation d´eau potable (0.2 mg/L), sauf dans 1seul échantillon d'eau de puits avec la valeur détectée de chlore résiduel de 1.6 mg/L. Les valeurs du pH mesurées dans les eaux des puits analysées ont variée entre 6.9 et 8.2 avec une moyenne de 7.55 ± 0.65. La température mesurée dans les eaux des puits analysées a variée de 12 à 28°C avec une moyenne de 20 ± 8°C.

## Discussion

L´objectif commun de l´industrie agroalimentaire et des gouvernements a consisté à prévenir la contamination des aliments par l'eau au moyen de la surveillance et du contrôle de la qualité de ce dernier. Ce contrôle nous a permis, grâces à des analyses bactériologiques périodiques, de connaître en permanence la potabilité de l´eau utilisée et surtout de quantifier certains germes qui peuvent avoir des incidences sur la qualité du produit fini et par conséquent sur la santé humaine. Notre étude a consisté à analyser les échantillons d'eau utilisée par les entreprises agroalimentaires du Nord du Maroc pour évaluer leur qualité bactériologique.

**Analyse bactériologique *Analyse de l'eau de réseau public utilisée par les entreprises agroalimentaires:*** les résultats ont révélé la bonne qualité bactériologique de l'eau du réseau public utilisée par les 15 industries agroalimentaires. Ces résultats ont prouvé que l'eau distribuée dans le réseau public est de bonne qualité. C'est la raison pour laquelle la majorité des entreprises agroalimentaires se sont contentées du contrôle de l'eau seulement aux points d'utilisation (sortie) et ont négligées le contrôle à l'entrée de l'usine. Les entreprises d'industrie agroalimentaire qui ont fait le contrôle de leur eau à l'entrée et à la sortie du réseau de l'usine devaient satisfaire les exigences du système de gestion HACCP qu'elles suivent. Selon les normes, le contrôle de l'eau du réseau public doit se faire chaque trimestre et d'une façon régulière. Au cours de cette étude, nous avons constaté que les entreprises procédant aux contrôles réguliers sont les entreprises satisfont les exigences du système de gestion HACCP ou elles ont été en cours de préparation à la mise en place d'un système qualité exigées actuellement pour accéder aux marchés internationaux.

***Analyse de l'eau de puits utilisée par les entreprises agroalimentaires:*** l'analyse bactériologique de l'eau de puits utilisée par différentes entreprises agroalimentaires y compris les fermes avicoles a montré que 40% des puits ont été contaminés. Cette contamination a concerné les MOR qui sont les indicateurs de la contamination globale et les CT, CF, E qui sont des indicateurs de la contamination fécale. Ces germes indicateurs de la pollution fécale ont été détectés le plus souvent pendant la saison sèche (Juin, Juillet et Aout) et la saison des pluies (Octobre, Novembre, Décembre et Janvier). Il a été observé une saison d´averse définie qui pourrait avoir indiqué son effet sur la qualité de l´eau bactériologique pendant la période d´étude. En effet, la contamination pendant la période pluviale a pu être due aux ruissellements des eaux de pluies ce qui fait augmenter la concentration bactérienne des eaux souterraines [4, 5] La contamination pendant les saisons sèches pourrait être attribuée à plusieurs facteurs tels que la température très élevée remarquée au cours de la période d'étude semble avoir un effet sur la dynamique des communautés bactériennes [6]; La diminution des niveaux d'eau souterraine pendant la saison sèche dans nombreux points ce qui cause l'augmentation de la concentration de ces différents germes. En plus, les conditions de puisage non hygiéniques chez la majorité des fermes avicoles ont favorisé leurs pollutions microbiennes. Nous avons constaté un degré élevé des coliformes par rapport aux Entérocoques. Ceci s'explique par le fait que les coliformes sont des bactéries d'origine fécale et environnementale. Les valeurs élevées en coliformes fécaux ont été de même enregistrées au niveau des eaux de puits de la nappe phréatique M'nasra Maroc [7].

Les bactéries entérites notamment les formes sporulées des sulfito-réducteurs (*Clostridium perfringens*), qui témoignent une contamination fécale ancienne en raison de leur aptitude d'être plus persistantes en milieu aquatique, ont été absentes dans les puits analysés car elles sont moins abondantes dans la flore intestinale des humains et des animaux par rapport aux coliformes [5, 8–10]

Concernant les MOR qui sont des indicateurs de contamination globale, leur dénombrement (à 22°C et 37°C) a été très modeste. Ceci pourrait être dû à l'hétérogénéité du groupe de bactéries constituant les MOR car les conditions du développement de certaines bactéries défavorisent la poussé des autres [11]. En plus, un éventuel choc thermique lors de l'incorporation en milieu gélosé (résultant du fait que la gélose est à une température de 45°C au moment de l'incorporation), pourrait entraîner une baisse de la viabilité bactérienne et, par conséquent, une sous-estimation du dénombrement [1] La contamination des puits dans les régions rurales a été attribuée au manque d'hygiène. En effet, la majorité des puits ont été non aménagés, ils ont été munis ou non de margelle en béton mais tous à ciel ouvert et dépourvus de couvercle qui constitue un dispositif de sécurité et de protection de la ressource. Ces conditions ne sont pas en accord avec l'Article 53 de La loi n°10-95 sur l'eau publiée en 1995 par le Ministère Délégué auprès du ministre de l'Energie, des Mines, de l'Eau et de l'Environnement, chargé de l'eau, disant que Tout système de distribution d´eau à ciel ouvert destinée à l´alimentation humaine est interdit [12]. Les abords conçus pour éviter toute stagnation de l'eau qui favorise le développement d'une flore dangereuse pour la nappe n'ont pas été aménagés. Par conséquent, la nappe d'eau a été facilement envahie par le ruissellement superficiel d'eau pluviale entraînant les eaux usées humains rejetées tout autour à la surface du sol ainsi que ces puits n'ont pas été traités par le chlore.

D'autre part, nos résultats ont montré que les germes pathogènes en particulier salmonelles n'ont pas été décelés. L'absence de ces germes pathogènes dans l'ensemble des puits analysés pourrait être due au fait que la teneur élevée en coliformes dans la plupart des puits peut exercer un effet compétitif voire inhibiteur sur la croissance de ce germe [5], que la quantité d'eau prélevée a été insuffisante par rapport au nombre relativement très faible de ces micro-organismes dans les eaux, ou que la technique adoptée n'a pas été assez fiable pour la culture de ce germe stressé dans ces eaux, on parle alors de bactéries « viables mais non cultivables » [13]. Ces bactéries bien que viables ne peuvent être dénombrées par les méthodes classiques. Cette perte de cultivabilité est le résultat de divers stress (stress nutritionnel, thermique, lumineux') que subissent les bactéries fécales lorsqu'elles sont rejetées dans un milieu aquatique naturel [13]. Par ailleurs, ces bactéries viables mais non cultivables peuvent conserver leur pathogénicité [14–17].

### Paramètres physicochimiques

Les valeurs de chlore résiduel ont respecté la norme établie par la législation d´eau potable (0.2 mg/L). Les germes de contamination (CT, CT, E, MOR) ont été détectés dans les eaux des puits non traités par le chlore. Selon les auteurs, la mauvaise qualité d´eau peut refléter la chloration inadéquate et aussi bien le stockage et le non traitement d´eau [5, 18] Les valeurs du pH mesurées dans les eaux des puits analysées ont variée entre 6.9 et 8.2 avec une moyenne de 7.55 ± 0.65. Ces valeurs n'ont pas variées pendant la période d'étude. Ainsi, le pH n'a pas influencé la variation saisonnière des microorganismes dans les eaux analysées. Même résultat a été montré au cours de l'étude des eaux des puits en brésil [5].

## Conclusion

Cette étude a permis de fournir des informations sur les principales sources d'eau utilisée par les industries agroalimentaires du Nord du Maroc et les facteurs associés à la qualité de l'eau utilisée par ces industries. Nous avons conclu que l'eau de réseau public utilisée par les entreprises agroalimentaires est une eau potable conforme aux normes en vigueur. Par contre la plupart des puits non traité se sont avérés contaminés par les germes indicateurs tels que les MOR, CT, CF, E. Cette pollution est généralement liée au non-respect des conditions de puisage hygiéniques ce qui est contradictoire à la règle marocaine. Tout programme de développement qui se veut complet doit prévoir des moyens pratiques et économiques pour assurer l'approvisionnement en eau potable et une stratégie volontariste en vue de réduire le risque de la transmission des bactéries véhiculées par l'eau.

### Etat des connaissances actuelle sur le sujet

- OMS donne une grande importance à la potabilité de l'eau utilisée dans le processus de la fabrication des denrées alimentaires est a mis en place des normes pour régulariser la potabilité de cette eau;
- Les pays industrialisés exigent l'accréditation des industries alimentaires en ISO 22000 pour le management de la sécurité des denrées alimentaires;
- Les industries agroalimentaires installent le système HACCP pour assurer la gestion de la qualité des processus de fabrication des aliments.

### Contribution de notre étude à la connaissance

- Donne une idée sur la qualité des eaux utilisées dans l'industrie alimentaire au Nord du Maroc;
- Montre l'importance du contrôle de la qualité de l'eau utilisée dans l'industrie agroalimentaire;
- Montre le degré du respect des industries agroalimentaires au règlement et aux normes juridiques.
